# Social support and depressive symptoms among physicians in tertiary hospitals in China: a cross-sectional study

**DOI:** 10.1186/s12888-021-03219-w

**Published:** 2021-04-29

**Authors:** Chang Fu, Guowen Wang, Xiuxin Shi, Fenglin Cao

**Affiliations:** 1 Department of Health Psychology, School of Nursing and Rehabilitation, Cheeloo College of Medicine, Shandong University, No. 44 Wenhuaxilu Rd, Jinan, 250012 Shandong China; 2 Department of Education, Shandong Provincial Hospital Affiliated to Shandong First Medical University, No. 324 Jingwuweiqilu Rd., Jinan, 250021 Shandong China; 3 Office of Medical Quality Control, Qilu Hospital, Cheeloo College of Medicine, Shandong University, No. 107 Wenhuaxilu Rd, Jinan, 250012 Shandong China

**Keywords:** Depressive symptoms, Physicians, Sex difference, Social support, Tertiary hospitals

## Abstract

**Background:**

Social support is an important factor for individual’s mental health. However, the association between social support and depressive symptoms among physicians in China’ tertiary hospitals has not been explored. This study aimed to investigate its association among physicians stratifying by sex.

**Methods:**

Six hundred fifty-six physicians were enrolled from 12 tertiary hospitals of Shandong Province, China. Depressive symptoms were assessed using the 10-item Center for Epidemiologic Studies Depression Scale. Social support was evaluated using the Social Support Rating Scale. Multiple linear regression analysis was used to examine the relationship between social support and depressive symptoms among physicians.

**Results:**

The prevalence of depressive symptoms was 42.3% and the average social support score was 38.82 ± 7.53 among physicians. Lower subjective social support scores (male: β = − 0.317, *p* < 0.001; female: β = − 0.241, *p* < 0.001) and lower objective social support scores (male: β = − 0.218, *p* = 0.038; female: β = − 0.277, *p* = 0.035) were associated with high depressive symptoms among physicians. Lower support utilization scores (β = − 0.472, *p* < 0.001) were associated with high depressive symptoms among male physicians.

**Conclusions:**

Chinese physicians had a higher prevalence of depressive symptoms and lower social support than the Chinese general population. Objective and subjective social support were inversely associated with depressive symptoms among male and female physicians while support utilization was inversely associated with depressive symptoms among male rather than female physicians. It is critical to improve physicians’ mental health through strengthening social support in China.

## Introduction

Depression has become one of the most common mental health problems worldwide, and it can significantly reduce the quality of life and workplace productivity [1]. Researchers have reported that, in the United States, 7.1% of adults have experienced at least one major depressive episode [2], and in Europe, the prevalence of depression is 14.8% [3]. In China, the prevalence of lifetime depression among the general population is 6.9%, and the 12-month prevalence of depression is 3.6% [4].

In recent years, the mental health of physicians has received increasing attention from researchers worldwide. Physicians are professionals who face high-level occupational stressors, such as long working hours, workload, and increasing professionalism-related demands in society [5]. As a result, they are vulnerable to experiencing depression [6]. Previous studies have demonstrated that the prevalence of depression among physicians is higher than the general population [7, 8]. As physicians are the direct providers of hospital services and a key element in the development of health services, depression among physicians is not only associated with their quality of life; but also hinders their professional performance and even influences the quality of healthcare [9]. Previous studies have identified several factors that influence depression among physicians, such as working hours [7] and age [8].

Social support refers to the social bonds, social integration, and primary group relations available to an individual [10]. It can be conveyed through verbal and nonverbal communication, and through actual or perceived exchanges of psychosocial or physical resources [11]. In recent years, the exploration of social support and its relationship with mental health has become one of the growing fields in psychology, particularly in the populations of older adults [12]. However, social support and mental health among physicians have received little attention. Previous studies revealed that social support is associated with depression among medical staff from developed countries [5, 13]. However, the sex differences have not been considered in these studies. Researchers found that female physicians have lower social status than male physicians and do not receive an adequate level of respect from nurses and patients [14, 15]. Thus, there may have sex differences among physicians in the relationship between social support and depressive symptoms.

Physicians in China may have heavier workloads than their counterparts in developed countries and have lower levels of social support than the Chinese general population. At the end of 2019, the number of physicians in China was 2.77 per 1000 population [16], which is lower than that in developed countries; for example, at the end of 2016, the number of physicians per 1000 population was 4.3 in Czech Republic, 4.0 in Italy, and 4.2 in Germany. In the process of healthcare reform, Chinese physicians have been asked to shoulder more responsibility for patient care, which may, in turn, have increased their workload [17]. In developed countries, physicians are usually afforded high social status and are well respected. However, in the past few years, the relationships between physicians and patients become intense and complex due to the occurrence of complications related to misdiagnosis and treatment [18]. Thus, increased stigmatization may lead to a lack of social support for physicians. However, there are few reports regarding both social support and depressive symptoms among Chinese physicians.

In China, tertiary hospitals are dedicated to providing medical care of patients with critical illness or complex diseases. Physicians in tertiary hospitals face more work stress than those in primary and secondary hospitals [19]. According to the Chinese Physician Practice Situation White Paper [20], the average working time for physicians in tertiary hospitals was 51 h per week (exceeding China’s legal limits of 40-h of work per week in China). We hypothesize that physicians in tertiary hospitals may have a high prevalence of depressive symptoms.

To maintain physicians’ mental health, it is critical to investigate the prevalence of depressive symptoms, the level of social support, and their association among physicians in tertiary hospitals. Additionally, to better understand the association between social support and depressive symptoms among physicians, sex differences should be taken into consideration. Thus, we conducted a survey of social support and depressive symptoms among a sample of physicians in China’s tertiary hospitals. This study will contribute to our understanding of both the level of social support and depressive symptoms among physicians and emphasizes the importance of actions required to improve the level of social support and to reduce depressive symptoms in this particular population.

## Methods

### Aim

The first aim of this study was to investigate the prevalence of depressive symptoms among physicians in tertiary hospitals in China. The second aim was to explore physicians’ level of social support. The third aim was to determine the relationship between social support and depressive symptoms among physicians, taking sex differences into account.

### Study design, sample, and procedures

This cross-sectional study was carried out from 30 July to 30 September 2020 in Shandong Province, China. Shandong is an eastern coastal province of China, with a geographic area of 157,900 km^2^. Shandong Province has sixteen prefecture-level cities, its total population reached 100.7 million, and its Gross Domestic Product (GDP) exceeded one trillion US dollars by the end of 2019. It is claimed that Shandong Province is a typical province in terms of its population structure, social and culture aspects [21]. A multistage random sampling method was used. First, according to per capita GDP, 16 cities in Shandong Province were classified into three groups: high, medium, and low. Two cities were randomly selected from each group. Second, two tertiary hospitals were selected in each city. Third, two-thirds of the departments such as the internal medicine, surgery, obstetrics and gynecology, pediatrics, and emergency departments were selected from each sample hospital. A total of 1000 physicians in the sample departments were invited to take part in the survey. The inclusion criteria for the participants were that they were registered physicians, employed by the respective hospital, and voluntarily agreed to participate. Excluded participants included those who were on vacation or who were on temporary leave for research or career development, or who suffered from serious mental or physical disorders that may have hindered their participation. The questionnaire was developed using “*Wenjuanwang*” (https://www.wenjuan.com/), which is a platform for the electronic questionnaires. We sent a web page related to the questionnaire survey to participants’ mobile phones by using *WeChat* (a social media app). Of 1000 physicians in tertiary hospitals, 677 physicians participated in the survey. After excluding participants with missing data, we included 656 participants in our analysis. The effective response rate was 96.89%.

### Measurements

#### Depressive symptoms

Depressive symptoms were assessed using the 10-item Center for Epidemiologic Studies Depression Scale (CES-D 10). The CESD-10 was highly validated for use in Chinese general populations and demonstrated adequate validity and acceptable reliability in previous studies [22, 23]. Four options were used to answer the 10 items: 0 = *rarely,* 1 = *some days (1–2 days),* 2 = *occasionally (3–4 days)*, and 3 = *most of the time (5–7 days)*. The participants’ answers were recorded as 0 (*rarely*) to 3 (*most of the time*) for the negatively scored questions and as 3 (*rarely*) to 0 (*most of the time*) for the positively scored questions. The total score ranges from 1 to 30, with higher scores indicating high depressive symptoms [23]. A cut-off point of 10 was recommended for this scale as indicating high depressive symptoms [24]. In the present survey, the Cronbach’s alpha for CES-D 10 scale was 0.70 in male group and 0.70 in female group.

#### Social support

Social support was measured using the Chinese version of the Social Support Rating Scale (SSRS), which has been used in a wide range of studies on Chinese population due to its high reliability and validity [25]. This 10-item scale consists of three dimensions: objective support (three items), subjective support (four items), and utilization of support (three items). The questions relating to objective support were Q2. What was your living situation in the past year?; Q6. What are your sources of financial support, and what helps you solve practical problems when you are in an emergency?; and Q7. What are your sources of comfort and concern when you are in an emergency? The questions regarding subjective support were Q1. How many friends do you have and how much support and help can they provide you?; Q3. How about your relationship with neighbors?; Q4. How about your relationship with colleagues?; and Q5. How much support and care have you received from family members? The questions focusing on utilization of support were Q8. How do you talk about trouble? Q9. How do you seek for help when you have trouble?; and Q10. How often do you participate in activities organized by groups (such as party organizations, religious organizations, labor unions, etc.)?. For Q1-Q4 and Q8-Q10, the respondent selects only one option for each item, scoring 1–4 points for options A, B, C, and D, respectively. Q5 is divided into five subitems (spouse or lover, parents, child/children, siblings, and other family members). For each subitem, the response “none” scores 1 point; “rare,” 2 points; “general,” 3 points; and “full support,” 4 points. For Q6 and Q7, the answer “no source” scores 0 points; otherwise, each source listed scores 1 point. The total SSRS score is the sum of the scores of the three subscales. A higher score indicates a higher level of social support [10]. In the present survey, the Cronbach’s alpha for SSRS scale was 0.81 in male group and 0.80 in female group.

#### Other variables

The demographic characteristics of the survey included age, sex, marital status (married or single), educational level (college diploma, bachelor’s degree, master’s degree or higher), and monthly income. The professional characteristics comprised departments (surgical or nonsurgical), professional titles (primary, intermediate, senior), employment status (formal or contract employee), and working hours per week. Lifestyle factors included cigarette smoking (current/past, never) and alcohol consumption (current/past, never). Health status was assessed by self-rating on a five-point scale (very poor, poor, fair, good, and very good). Self-rated health was categorized as good (including very good and good), fair, or poor (including poor and very poor).

#### Statistical analysis

The data were analyzed using the Statistical Package for the Social Sciences (SPSS) version 20.0 (SPSS Inc., Chicago, IL, USA). Normal distribution of continuous variables was analyzed using the Kolmogorov-Smirnov test, Histogram plot, and P-P plot, which showed that they were normally distributed. Variance Inflation Factor (VIF) values larger than 10 were regarded as having presence of multicollinearity [26], and VIF of all independent variables in our study<10. Reliability was assessed with Cronbach’s alpha. The characteristics of the overall respondents were described using means and standard deviations (*SD*) for continuous data and percentages for categorical data. We divided our sample into two groups: male and female physicians. A comparison between groups was performed using independent *t*-tests for continuous variables and χ^2^-tests for categorical variables. Multiple linear regression analysis was used to examine the association between social support (objective social support, subjective social support, and support utilization) and depressive symptoms stratified by sex. Regression coefficient estimates (β), standard error (SE) of β, 95% confidence intervals (CI) of β, and *p*-values were analyzed. The dependent variable was depressive symptoms. Potential confounders that were added into the multiple linear regression models were identified either through literature, univariate regression analysis, or Change-in Estimate procedures [27]. Statistical significance was set at *p-*value<0.05.

### Ethical considerations

The Ethical Review Committee of the School of Nursing and Rehabilitation, Shandong University, approved the current study (approval number: 2020-R-50). All subjects gave their informed consent for inclusion before participating in the survey.

## Results

### Sample characteristics

Table 1 presents the characteristics of the participants. Of the 656 participants (mean age = 37.24 years, *SD* = 9.47 years), 54.5% were men, 80.5% were married, 55.5% had received a master’s degree or higher, and 58.2% reported having a fair health status. Professionally, 53.0% were working in surgical departments, 43.9% had a primary professional title, and 54.6% were formal employees. More than three-quarters of the participants were non-smokers (83.2%), and 41.5% did not consume alcohol. Of the participants, 40.7% earned between ¥ 5000 and ¥ 8000 Renminbi (RMB) per month, and 94.4% worked more than 40 h per week. The mean score of CES-D 10 score was 8.97 (*SD* = 5.40). Additionally, using the 10-item cut-off point, about 42.3% of the participants were found to have probable depression (male: 40.1%; female: 44.8%). There were statistically significant differences in age, marital status, monthly income, department, professional status, cigarette smoking, and alcohol consumption between male and female physicians (*p* < 0.05).

**Table 1:** Characteristics of the participants according to a sex comparison

Characteristics	Total Sample (*n* = 656)	Male (*n* = 357)	Female (*n* = 299)	*χ*^*2*^*/t*	*p-*value
Age, years, mean ± SD	37.24 ± 9.47	38.80 ± 9.76	35.38 ± 8.76	4.731	**< 0.001**
Marital status, n(%)				7.300	**0.007**
Married	528 (80.5)	301 (84.3)	227 (75.9)		
Single	128 (19.5)	56 (15.7)	72 (24.1)		
Educational level, n(%)				0.417	0.812
College diploma	18 (2.7)	10 (2.8)	8 (2.7)		
Bachelor’s degree	274 (41.8)	153 (42.9)	121 (40.5)		
Master’s degree or higher	364 (55.5)	194 (54.3)	170 (56.9)		
Monthly income(¥, RMB), n(%)				14.715	**0.002**
<5000	140 (21.3)	65 (18.2)	75 (25.1)		
5000-8000	267 (40.7)	136 (38.1)	131 (43.8)		
8000-10,000	128 (19.5)	87 (24.4)	41 (13.7)		
>10,000	121 (18.4)	69 (19.3)	52 (17.4)		
Department, n(%)				26.248	**< 0.001**
Surgical	348 (53.0)	222 (62.2)	126 (42.1)		
Non-surgical	308 (47.1)	135 (37.8)	173 (57.9)		
Professional status, n(%)				17.817	**< 0.001**
Primary	288 (43.9)	133 (37.3)	155 (51.8)		
Intermediate	191 (29.1)	107 (30.0)	84 (28.1)		
Senior	177 (27.0)	117 (32.8)	60 (20.1)		
Employment status, n(%)				1.656	0.198
Formal employee	358 (54.6)	203 (56.9)	155 (51.8)		
Contract employee	298 (45.4)	154 (41.3)	144 (48.2)		
Cigarette smoking, n(%)				91.712	**< 0.001**
Current/past	110 (16.8)	106 (29.7)	4 (1.3)		
No	546 (83.2)	251 (70.3)	295 (98.7)		
Alcohol consumption, n(%)				228.635	**< 0.001**
Current/past	384 (58.5)	304 (85.2)	80 (26.8)		
No	272 (41.5)	53 (14.8)	219 (73.2)		
Working hours per week, n(%)				1.716	0.633
≤ 40	37 (5.6)	18 (5.0)	19 (6.4)		
41–50	192 (29.3)	105 (29.4)	87 (29.1)		
51–60	189 (28.8)	98 (27.5)	91 (30.4)		
>60	238 (36.3)	136 (38.1)	102 (34.1)		
Self-rated health, n(%)				1.028	0.598
Good	204 (31.1)	117 (32.8)	87 (29.1)		
Fair	382 (58.2)	203 (56.9)	179 (59.9)		
Poor	70 (10.7)	37 (10.4)	33 (11.0)		
Depression scale, mean ± SD	8.97 ± 5.40	8.84 ± 5.36	9.12 ± 5.56	−0.661	0.509
Depressive symptoms, n(%)				1.511	0.219
Yes	277 (42.2)	143 (40.1)	134 (44.8)		
No	379 (57.8)	214 (59.9)	165 (55.2)		

1 USD ≈ ¥ 6.96 RMB (August 2020 exchange rate). *Abbreviations*: *RMB* renminbi, *SD* standard deviation

### The differences between social support scores among participants and norms of the Chinese general population

As Table 2 shows, the mean total social support score was 38.82 (*SD* = 7.86). Scores on all three dimensions of social support and the total social support score in our study were lower than the norms of the Chinese general population [28, 29]. There were significant differences in the objective social support score, the support utilization score, and the total social support score between this study and the norms of the Chinese general population (*p* < 0.05).

**Table 2:** The difference of social support scores among participants and the norms of Chinese general population

Variables	Total sample (*n* = 656)	Norms of general population	*t*	*p-*value
Subjective supports core, mean ± SD	23.59 ± 5.21	23.81 ± 4.75	−1.079	>0.050
Objective social support score, mean ± SD	7.87 ± 2.54	12.68 ± 3.47	−48.428	**< 0.001**
Support utilization score, mean ± SD	7.36 ± 1.91	9.38 ± 3.40	−27.046	**< 0.001**
Total social support score, mean ± SD	38.82 ± 7.86	44.38 ± 8.38	−18.067	**< 0.001**

*Abbreviations*: *SD* standard deviation

### The differences between social support scores among male and female physicians

As Table 3 shows, There were no statistically significant differences in scores of subjective support, objective social support, and the total social support score of male and female physicians. Support utilization score of female physicians was significant higher than male physicians (*P* < 0.05).

**Table 3:** The differences of social support scores of male and female physicians

Variables	Male (*n* = 357)	Female (*n* = 299)	*t*	*p-*value
Subjective support score, mean ± SD	23.87 ± 5.18	23.26 ± 5.23	1.497	0.135
Objective social support score, mean ± SD	7.90 ± 2.57	7.84 ± 2.50	0.269	0.788
Support utilization score, mean ± SD	7.18 ± 1.94	7.57 ± 1.86	− 2.598	**0.010**
Total social support score, mean ± SD	38.94 ± 7.90	38.67 ± 7.81	0.452	0.652

*Abbreviations*: *SD* standard deviation

### Multiple linear regression analysis

Table 4 shows the associations between social support and depressive symptoms. After adjusting for all covariates, the results of the multiple linear regression analysis showed that, among male physicians, a lower subjective support score was associated with high depressive symptoms (β = − 0.317, SE = 0.058, *p* < 0.001), a lower objective support score was associated with high depressive symptoms (β = − 0.218, SE = 0.105, *p* = 0.038), and lower support utilization score was associated with high depressive symptoms (β = − 0.472, SE = 0.129, *p* < 0.001); Among female physicians, a lower subjective support score was associated with high depressive symptoms (β = − 0.241, SE = 0.065, *p* < 0.001) and lower objective support score was associated with high depressive symptoms (β = − 0.277, SE = 0.131, *p* = 0.035). There was no significant correlation between support utilization score and depressive symptoms among female physicians.

**Table 4:** The association between social support and depressive symptoms stratified by sex

Variables	Male physicians			Female physicians		
	Β (SE)	95% CI of β	*p-*value	β (SE)	95% CI of β	*p-*value
Subjective supports score	−0.317 (0.058)	− 0.431 ~ − 0.204	**< 0.001**	−0.241 (0.065)	− 0.370 ~ − 0.113	**< 0.001**
Objective supports score	−0.218 (0.105)	− 0.424 ~ − 0.013	**0.038**	−0.277 (0.131)	− 0.532 ~ − 0.014	**0.035**
Support utilization score	− 0.472 (0.129)	−0.726 ~ − 0.218	**< 0.001**	−0.102 (0.162)	− 0.425 ~ 0.214	0.528
Age	−0.026 (0.049)	−0.122 ~ 0.070	0.596	−0.017 (0.059)	− 0.135 ~ 0.098	0.780
Marital status (ref. Married)						
Single	1.572 (0.769)	0.060 ~ 3.084	**0.042**	1.729 (0.810)	0.134 ~ 3.328	**0.034**
Education level (ref. college diploma)						
Bachelor’ degree	1.247 (1.427)	−1.561 ~ 4.055	0.383	−2.354 (1.731)	−5.578 ~ 1.066	0.175
Master’ degree or higher	1.882 (1.489)	−1.047 ~ 4.812	0.207	−3.779 (1.786)	**−7.314 ~ −0.275**	**0.035**
Monthly income(¥, RMB) (ref. < 5000)						
5000-8000	0.265 (0.698)	−1.108 ~ 1.639	0.704	−0.233 (0.727)	−1.656 ~ 1.212	0.749
8000-10,000	−0.984 (0.825)	−2.608 ~ 0.639	0.234	0.026 (1.020)	−1.996 ~ 2.027	0.980
>10,000	−2.406 (0.933)	−4.241 ~ −0.572	**0.010**	−0.113 (1.074)	−2.239 ~ 1.995	0.917
Cigarette Smoking (ref. Current/Past)						
Never	−0.476 (0.503)	−1.466 ~ 0.514	0.345	−6.703 (2.278)	−11.162 ~ −2.181	**0.004**
Alcohol consumption (ref. Current/Past)						
Never	0.357 (0.651)	−0.925 ~ 1.638	0.584	−1.036 (0.587)	−2.231 ~ 0.092	0.079
Department (ref. Surgical)						
Non-surgical	−0.382 (0.470)	−1.306 ~ 0.542	0.417	0.500 (0.532)	−0.574 ~ 1.527	0.348
Professional title (ref. Primary)						
Intermediate	1.855 (0.720)	0.439 ~ 3.270	**0.010**	−1.385 (0.773)	−2.873 ~ 0.185	0.074
Senior	1.766 (1.087)	−0.372 ~ 3.905	0.105	−1.311 (1.320)	−3.881 ~ 1.323	0.322
Employment status (ref. Formal employee)						
Contract employee	0.024 (0.566)	−1.090 ~ 1.137	0.967	−0.064 (0.660)	−1.384 ~ 1.218	0.923
Weekly working hours (ref. ≤40)						
41–50	−1.140 (1.092)	−3.288 ~ 1.009	0.297	2.014 (1.152)	−0.230 ~ 4.314	0.082
51–60	0.079 (1.120)	−0.589 ~ 1.642	0.944	2.830 (1.164)	0.531 ~ 5.117	**0.016**
>60	−0.447 (1.135)	−2.679 ~ 1.785	0.694	3.383 (1.148)	1.123 ~ 5.649	**0.003**
Self-rated health (ref. good)						
Fair	2.681 (0.517)	1.664 ~ 3.697	**< 0.001**	2.645 (0.603)	1.418 ~ 3.805	**< 0.001**
Poor	5.421 (0.834)	3.780 ~ 7.062	**< 0.001**	6.316 (0.985)	4.327 ~ 8.218	**< 0.001**
△F	13.255			9.093		
R^2^	0.454			0.408		
Adjusted R^2^	0.420			0.363		

*Abbreviations*: *β* the coefficients, *CI* confidence interval, *SE* standard error, *RMB* renminbi

## Discussion

To our knowledge, this is the first study to investigate the prevalence of depressive symptoms and the association between depressive symptoms and levels of social support among Chinese physicians in tertiary hospitals. The present study will provide useful data for health policy-makers, hospital administrators, and society to take effective measures to further promote physicians’ mental health.

In the present study, the prevalence of depressive symptoms among physicians was 42.2% (male: 40.1%; female: 44.8%), which was higher than the Chinese general population (12.6%) [30]. Using the same measurement of depressive symptoms as in our study, the prevalence of depressive symptoms was 33.08% among older adults [31] and was 28.1% among adolescents [32] in China. Despite the differences in participants’ age, gender, and place of residence, when compared to the Chinese general population, our results showed that the prevalence of depressive symptoms among physicians in tertiary hospitals is significantly higher. Additionally, our results showed that the prevalence of depressive symptoms among Chinese physicians was higher than that reported in other studies from developed countries. Using the same measurement, the prevalence of depressive symptoms was 28% among physicians in Japan [3]. A systematic review and meta-analysis found that 28.8% of resident physicians have depression or depressive symptoms in the United States [33]. The relatively high prevalence of depressive symptoms among physicians in tertiary hospitals in China may be attributable to various factors. First, compared to physicians in developed countries, Chinese physicians have lower income, longer working hours, and heavier workloads. The socioeconomic status of Chinese physicians is lower and there is arguably a mismatch between workload and reward [34]. In our study, more than half of the physicians had a monthly income of less than ¥8000 (RMB), approximately $1149 (USD), and 65.2% of physicians worked more than 50 h per week, including 36.3% who worked more than 60 h despite the fact that the legal limit for working hours per week in China is 40 h. According to the Medscape Physician Compensation Report 2020 [35], the average annual compensation for a physician in the United States was $341,000 (USD), about $28,417 (USD) per month, and the average number of working hours per week was 53.4 h. The lower income and longer working hours could lead to increased depressive symptoms among Chinese physicians [36]. Second, compared to other professionals, medical staff are more likely to work in an intense workplace with weaker administrative measures, which may hinder their professional success or self-fulfillment [21]. In most hospitals, the professional promotion parameters usually emphasize the publications of scientific papers, funding, and awards. The professional skills and achievements are less likely to be considered [37]. However, most physicians often have heavy workloads and do not have sufficient time to engage in scientific research, which puts them at a disadvantage with regard to promotion. The difference among personal desires, goals, and reality may cause psychological stress among medical professionals [21] and may increase their depressive symptoms. Third, there was a significant difference in social status among Chinese physicians in comparison to those in developed countries; for example, there were some negative reports related to physicians’ clinical practice in the past few years [15]. These may have a negative impact on psychological pressure and depressive symptoms among Chinese physicians [38]. Other influencing factors of high prevalence of depressive symptoms among physicians in China are worthy of further research.

Our results showed that physicians’ total social support score was significantly lower than the Chinese general population. Regarding the three dimensions of social support, both objective social support and support utilization among physicians were significantly lower than the Chinese general population; only subjective social support was not. In the SSRS, objective social support refers to any kind of visible or actual social support, which is usually provided by family members, the government, local communities, and nongovernmental organizations. The lower social status of Chinese physicians may limit their access to objective social support from others, beyond family members. In addition, physicians often have long working hours and they have few time to participate in social activities. Support utilization represents the utilizing degree of the social support available. Previous studies have found that physicians are often reluctant to seek help because of their overconfidence [39]. Apart from this reason, we assume that physicians often work excessive hours and have few vacation days; thus, they have less time to join in social interactions, which may weaken their social support networks [40].

In our study, there is no difference in objective social support between male and female physicians. The explanation may be that both male and female physicians have longer working hours, fewer time to participate in social organizations, which may limit their access to objective social support from society. We found that there is no difference in subjective social support between male and female physicians. From the present study, we found that there was no statistically significant differences in educational levels between the male and female physicians. In China, higher education degree means a principal definer of social status [41]. Thus, female physicians usually think they may have equal social status as male physicians and receive similar support from society. Our results showed that support utilization score among female physicians was significant higher than male physicians. This phenomenon may be explained by the issue of the traditional masculine norms. The social construction of masculinity including competitiveness, self-reliance, and less emotional express, may influence male’s help-seeking behavior, while women are more likely than men to seek help from others for problems such as depression, physical discomfort, and stressful life events [42, 43].

Our findings revealed that subjective social support was inversely associated with depressive symptoms among Chinese physicians. Physicians tended to receive higher levels of subjective social support were less likely to have depressive symptoms. Subjective social support as a term for individuals’ experience or feelings of social support, represents personal emotional experience and satisfaction with being supported, understood, and respected by others. Subjective social support helps individuals establish a positive self-image, self-efficacy, and higher self-esteem, which acts as protective factors against depressive symptoms [44, 45]. Moreover, a higher subjective support score means a better understanding of self-monitoring health status [46], which could have a positive impact on the reduction of depressive symptoms among physicians.

Objective social support refers to any type of visible or actual social support that may reduce work related stress [47]. It may help physicians buffer the effects of heavy workloads and maintain a better mental health status and well-being. In the present study, the findings showed that physicians with higher objective social support score were less likely to have depressive symptoms. Higher objective social support scores usually represents that they may receive more support from family members, the government, local communities, and nongovernmental organizations. Additionally, a friendly social environment is related to better mental health of individuals [48].

According to our data, male physicians with higher support utilization score were less likely to have depressive symptoms. Support utilization reflects the degree to which an individual proactively seeks help and social interaction. Higher support utilization indicated that individuals are more likely to seek help from family members, friends, social organizations, and society. From our data, male physicians with higher support utilization score were less likely to have depressive symptoms, which was consistent with the findings that a better connection with others can alleviate the harmful impact of stressful life events [10]. Another explanation is that individuals who participate in social interactions often have extensive social networks and more chances to access various forms of health-related information [49]. Encouraging physicians to widen their social networks may be useful to protect them from having depressive symptoms.

Interestingly, although female physicians had higher support utilization score than male physicians, there was no significant association between support utilization and depressive symptoms in our data. Female physicians’ quality of social networks and family role may explain this phenomenon. The support from supervisors and colleagues are more important than that from family and friends in workplace [50]. Previous studies revealed that in comparison to male physicians, female physicians often have poor coworker relationships, get less respect, and do not obtain sufficient help from nurses and their male colleagues [51, 52]. Additionally, female physicians often shoulder the responsibility of taking care of their own families, including raising children and caring for the elderly and have less leisure time than their male colleagues [53], which can reduce their time and the quality of interaction with friends. Thus, the beneficial effects of support utilization on depressive symptoms may be limited among female physicians.

Our findings also showed that being single and having poor self-rated health status were associated with high depressive symptoms among physicians, which was consistent with the results from a previous study [7]. Non-smokers had low depressive symptoms among female rather than male physicians, which was also consistent with the previous report that cigarette smoking has more harmful impact on woman’s mental health [54]. From our findings, higher income was associated with low depressive symptoms only among male physicians while longer working time was associated with high depressive symptoms only among female physicians. A possible explanation for these phenomena is the gender differences in family’s role. Women usually need to deal with dual pressures from work and family [14], while men have an advantage in the labor market [55]. Thus, men are assumed to play predominant role as financial providers. Those who earn less often have low self-esteem and high depressive symptoms [56]. While women including physicians usually spend more time on housewifery than their male counterparts [15]. Therefore, female physicians may be exhausted by long working time and housework load [57], resulting in high depressive symptoms. Several studies about the educational level with high depressive symptoms were conducted, but the results are still mixed [58, 59]. Our study revealed that higher educational level was associated with low depressive symptoms only among female physicians. It is suggested that higher educational level elevate their self-esteem and professional position, which can reduce the gender inequality and depressive symptoms [52, 56]. Our data showed a higher prevalence of depressive symptoms existed in male rather than female physicians with intermediate professional title, further study is needed to explain this phenomenon.

This study has several limitations. First, this was an online self-reporting questionnaire survey, so there was a risk of recall bias as well as selection and observation bias, which could result in inaccurate responses. Second, our study was a cross-sectional study, thus, the causal relationship between social support and depressive symptoms among physicians could not be determined. Though the present survey was conducted while progress has been made in containing COVID-19 and restrictions are gradually relaxed in China, the impact of the COVID-19 epidemic on physicians’ mental health needs to be considered [60].

### Policy implications

Based on our findings, we suggest that policy-makers and health administrators should (1) provide comprehensive counseling and support services for physicians to help them reduce depressive symptoms, regardless of gender; (2) improve the quality of social support and establish a harmonious coworker relationships for physicians, especially for female physicians; (3) reduce physicians’ working hours and workloads, (4) increase physicians’ income; (5) develop educational programs aimed to alleviate physicians’ depressive symptoms. (6) take effective measures to prohibit stigma-related events toward medical staff; (7) optimize the current professional title promotion system.

## Conclusion

Our study revealed that there was a higher prevalence of depressive symptoms and lower social support among male and female physicians in tertiary hospitals with comparison to the Chinese general population. Among both male and female physicians, higher scores for subjective social support and objective social support were associated with lower depressive symptoms. A higher support utilization score was associated with lower depressive symptoms only among male physicians. These findings signify that physicians’ mental health in China should be considered as an important issue by hospital administrators and healthcare policy makers. To improve physicians’ mental health through further strengthening social support and considering sex differences is critical.

## Data Availability

The datasets generated and/or analyzed during the current study are not publicly available due to agreements with participants who restricted data sharing but are available from the corresponding author on reasonable request.

## Abbreviations

B: the coefficients
CES-D 10: 10-item Center for Epidemiologic Studies Depression Scale
CI: Confidence interval
RMB: Renminbi
GDP: Gross domestic product
SD: Standard deviation
SE: Standard error
